# Public Health Interventions: Reaching Latino Adolescents via Short Message Service and Social Media

**DOI:** 10.2196/jmir.2178

**Published:** 2012-07-12

**Authors:** Amita N Vyas, Megan Landry, Marisa Schnider, Angela M Rojas, Susan F Wood

**Affiliations:** ^1^Maternal and Child HealthDepartment of Prevention and Community HealthGeorge Washington UniversityWashington, DCUnited States; ^2^Health BehaviorDepartment of Prevention and Community HealthGeorge Washington UniversityWashington, DCUnited States; ^3^Program EvaluationCassandra Drennon & AssociatesAthens, GAUnited States; ^4^Jacobs Institute for Women's HealthDepartment of Health PolicyGeorge Washington UniversityWashington, DCUnited States

**Keywords:** Public health interventions, SMS, short message service, social media, health behavior, Latinos, adolescents

## Abstract

**Background:**

Adolescents are substantial users of short message service (SMS) and social media. The public health community now has more opportunities to reach this population with positive youth development and health messages through these media. Latinos are a growing and youthful population with significant health risks and needs. This population may benefit from SMS and social media health interventions.

**Objective:**

To examine (1) SMS and social media utilization and behavior among Latino youth, and (2) how SMS and social media can be effectively used as a component of public health interventions focused on decreasing sexual risk taking among Latino youth.

**Methods:**

A mixed-methods approach, using both quantitative survey data and qualitative interview data, was used to provide a robust understanding of SMS and social media use and behavior for public health interventions. We recruited 428 ninth and tenth grade, self-identifying Latino adolescents to participate in a quantitative survey. Additionally, we conducted five key informant interviews with staff and 15 youth.

**Results:**

We found that 90.8% (355/391) of respondents had access to a mobile phone either through having their own or through borrowing or sharing one. Of those who had access to a mobile phone, 94.1% (334/355) used SMS, with 41.1% (113/275) sending and receiving more than 100 text messages per day. Of 395 respondents, 384 (97.2%) had *at least one *social media account, and the mean number of accounts was 3.0 (range 0–8). A total of 75.8% (291/384) of adolescents logged in to their account daily. Of those with a social media account, 89.1% (342/384) had a Facebook account. Youth who took the survey in English were significantly more likely than those who took it in Spanish to have access to a mobile phone (χ^2^_1 _= 5.3; 93.3% vs 86.3%; *P *= .02); to be high-volume texters (χ^2^_2 _= 16.8; 49.4% vs 25.3%; *P *< .001); to use the Internet daily (χ^2^_1 _= 5.0; 76.6% vs 66.0%; *P *= .03); to have a Facebook account (χ^2^_1 _= 9.9; 90.9% vs 79.7%; *P *= .002); and to have a greater mean number of social media accounts (*t*_387 _= 7.9; 3.41 vs 2.07; *P *< .001).

**Conclusions:**

SMS and social media are pervasive among Latino youth. Program staff and youth perceive these as credible and essential methods of communication in the context of public health programs. Public health interventions must continue to innovate and maximize new ways to reach young people to reinforce public health messages and education.

## Introduction

The rapid expansion of access to and reach of mobile technology among adolescents gives the public health community new opportunities for delivering critical and timely interventions to young people that can promote positive behavior change. It is projected that by 2025, one-quarter of the adolescent US population will be Latino [1] and that Latino youth are at greater risk for engaging in unhealthy sexual behaviors leading to sexually transmitted diseases and teen pregnancy [2,3]. Nationally, the Latino community has both higher teen pregnancy and higher birth rates than the overall US population, with 53% of Latinas getting pregnant in their teens, approximately twice the national average [1]. Although dozens of evidence-based programs aimed at reducing sexual risk taking have been identified nationally, effective behavioral interventions to reduce sexual risk taking among Latino adolescents are sparse. Thus, as mobile technology becomes increasingly adopted to promote health, the need for innovative programs dedicated to the Latino adolescent population that also incorporate short message service (SMS) and social media is significant [4-10].

Using SMS, also known as text messaging, for health promotion is particularly appealing for reaching individuals not regularly in contact with health services, and for behaviors that may be socially sensitive, such as sexual health and teen pregnancy, as they offer a confidential means of communication [11]. According to the Pew Hispanic Center, Latinos are high users of mobile technology, with 55% of Latino youth using text messages as their main method to communicate with friends [12].

In addition to using SMS, over 150 health promotion activities for sexual health use social media websites that target young people [13]. Most of these focus on sexual health in general or on the human immunodeficiency virus [14]. Facebook, Twitter, and MySpace are the most popular social media sites used for health promotion. Some of the capabilities that make these websites attractive for health promotion are public and private messaging, photo and video sharing, live updates, and the possibility of forming groups pages and including applications such as games and polls [14].

Social media, including SMS, to supplement a program, or as a stand-alone program, have been useful in improving health outcomes, increasing adherence to medication schedules and appointments, and improving patient–provider communication, health information communication, data collection, and access to health records [4,6,15]. Using SMS to reinforce key prevention concepts displayed in a program is important to broadening the effects a face-to-face intervention has on the program’s participants [15,16]. Periodic prompts and reminders have also been an effective method for encouraging and reinforcing healthy behaviors [4,17]. Increasing communication, accountability, and reinforcement created through SMS and social media can increase participants’ ability to remember changes they have learned in the program and that they should be making [4].

Although a handful of studies have focused on SMS and social media use among Latino youth, relatively few have focused on how this behavior may be integrated into public health interventions. This study used data from a larger ongoing research and demonstration project, Empowering Latino Youth Program (ELYP). The objective of this analysis was to examine (1) SMS and social media utilization and behavior among Latino youth, and (2) how SMS and social media can be effectively used as a component of public health interventions focused on decreasing sexual risk taking among Latino youth. This study was reviewed and approved by the George Washington University Internal Review Board (IRB# 011217).

## Methods

We used a mixed-methods approach, using both quantitative survey data and qualitative interview data, to provide a robust understanding of SMS and social media use and behavior for public health interventions.

### Quantitative Phase

#### Sample and Procedures

We recruited ninth and tenth grade, self-identifying Latino adolescents from 12 public high schools in Maryland, USA to participate in the ELYP. ELYP is an after-school intervention aimed at reducing sexual risk taking among Latino youth and includes both an intervention and a control arm. This is a demonstration project incorporating both quantitative and qualitative measures at baseline and three points of follow-up. The present study used baseline data from 428 youth participating in the first year of ELYP. Active parental consent was obtained prior to data collection, and youth assent was read and reviewed with youth prior to survey administration. To ensure privacy and reduce reporting bias, we administered surveys via individual laptop computers with audio-capability to youth with low literacy levels so that they could listen to each question and have it read to them if desired. Study participants had the choice to complete the survey in English or Spanish and were given US $10 gift cards for their efforts in completing the survey. On survey completion, the data were stored in an encrypted file to be read only by the survey design software (Snap Surveys Ltd, Portsmouth, NH, USA).

#### Instruments and Measures

We adapted most of the questions used in this study from the National Longitudinal Study of Adolescent Health [18], Youth Risk Behavior Survey [19], and the PEW Internet Research Center’s 2011 teen survey [20]. Our survey included demographic variables such as age, grade, gender, US-born or years in the United States, family structure, and parents’ educational level (Multimedia Appendix 1).

The survey asked a series of questions to assess SMS and social media behavior. Youth self-reported access, utilization, reasons for use, and frequency of using a mobile phone, texting, the Internet, and social media sites. The following response categories were used to assess frequency of overall *daily SMS*: 1–10 text messages per day, 11–20 per day, 21–50 per day, 51–100 per day, 101–200 per day, and more than 200 text messages per day. Finally, participants were asked on which social media sites they had an account: Facebook, MySpace, Twitter, Yahoo!, YouTube, myYearbook, Tumblr, Google Buzz, Flickr, Ustream, other, or “I don’t have my own profile or account on a social media site.”

#### Analysis

We used SPSS version 20 (IBM Corporation, Somers, NY, USA) for all statistical analyses. Variables were recoded and collapsed to increase the saliency of the data. Patterns of missing data were analyzed and, although data were missing for key variables, it was for less than 10% of respondents. Therefore, the data presented include all participants (n = 428), and we used pairwise deletion for the bivariate analysis to retain as many respondents as possible in the analysis. Quantitative results were analyzed to provide summary descriptive statistics and cross-tabulations. We calculated chi-square and *t *test statistics to examine differences in social media utilization by selected demographic characteristics including gender, US-born, and survey language. Only significant findings of the bivariate analysis are presented.

### Qualitative Phase

#### Sample and Procedures

The aim of the key informant interviews and youth triads was to assess staff and youth perceptions regarding feasibility of an SMS and Facebook component in the ELYP after-school program. The qualitative component consisted of (1) key informant interviews with staff delivering the *pilot *ELYP after-school program, and (2) youth triads with ninth and 10th grade Latino youth. We recruited and interviewed 5 staff members (3 women and 2 men). A convenience sample of 15 youth who were involved in the ELYP pilot after-school program were recruited and participated in the triads. Of the 15 youth in the triads, most were female. Youth who participated in the triads received incentives such as gift cards and food for their time and cooperation.

#### Instruments and Measures

We conducted key informant interviews using a semistructured field guide. The field guide contained 7 predetermined questions with probes. Each interview took approximately 45 minutes to complete (Multimedia Appendix 2). Youth triads were conducted using a semistructured field guide consisting of 13 questions with additional probes (Multimedia Appendix 3). The triads lasted between 45 and 90 minutes. We conducted two triads in Spanish and three in English.

#### Analysis

All interviews and triads were audio-recorded and transcribed verbatim, and 2 people from the research team read the staff interview and youth triad transcripts. The team convened before and after analyzing the transcripts to agree on a series of thematic codes to describe various categories and subcategories addressed throughout the interviews and triads. The codes were then entered and applied to the transcripts using NVivo software (version 9, QSR International Pty Ltd, Southport, UK).

## Results

### Quantitative Phase

The study sample consisted of 428 self-identifying Latino adolescents with a mean age of 15.7 years (Table 1). The sample included slightly more girls (244/428, 57.0%); the majority of participants were in ninth grade (247/428, 57.7%); 262/428 (61.2%) took the survey in English; and 176/428 (41.1%) lived with both parents and 160/428 (37.4%) with their mother only. Less than half (150/428, 35.1%) of the sample was born in the United States, with 47.1% (112/238) of those not born in the Unites States being recent immigrants (within 0–3 years). The parent’s educational level was quite low for both mother and father, with 26.6% (114/428) of mothers not having had any school or not finishing high school, and 34.3% (147/428) of fathers not having had any school or not finishing high school. Interestingly, we noted that a large percentage of youth were unsure or did not know their parent’s education level.

As Table 2 shows, 355/391 (90.8%) respondents had access to a mobile phone either through having their own or through borrowing or sharing one. Of those who had access to a mobile phone, 334/355 (94.1%) used SMS, with 113/276 (40.9%) sending and receiving more than 100 text messages per day. Youth reported using their mobile phones for a variety of reasons, including to take pictures, access the Internet, send or receive instant messages, play games, and send or receive email.

A total of 98.0% (385/393) of participants had access to the Internet (Table 2). Of those with access, 280/385 (72.7%) used the Internet daily and 330/385 youth use it for schoolwork (85.7%). Of 395 respondents, 384 (97.2%) had *at least one *social media account, and the mean number of accounts was 3.0 (range 0–8). In total, 75.8% (291/384) of adolescents logged in to their account daily. Of those with a social media account, 342/384 (89.1%) had a Facebook account with the next closest account being YouTube (205/384, 53.4%). Youth accessed social media to post photos or videos, to post comments, for instant messaging, and to update their status.

Table 3 presents SMS and social media behavior by US- versus foreign-born, survey language, and gender. As shown, girls were significantly more likely to use the Internet every day (χ^2^_1 _= 3.7; 76.2% vs 67.3%; *P *= .05) and on average had significantly more social media accounts than their male counterparts (*t*_386 _= 2.8; mean 3.11 vs 2.61; *P *< .01). With respect to nativity, US-born participants on average had significantly more social media accounts than foreign-born participants (*t*_355 _= –4.7; 3.41 vs 2.54; *P *< .001). Survey language is often a proxy measure for acculturation and yielded several significant findings. Youth who took the survey in English were significantly more likely than those who took it in Spanish to have access to a mobile phone (χ^2^_1 _= 5.3; 93.3% vs 86.3%; *P *= .02); to be high-volume texters (χ^2^_2 _= 16.8; 49.4% vs 25.3%; *P *< .001); to use the Internet daily (χ^2^_1 _= 5.0; 76.6% vs 66.0%; *P *= .03); to have a Facebook account (χ^2^_1 _= 9.9; 90.9% vs 79.7%; *P *= .002); and to have a greater mean number of social media accounts (*t*_387 _= 7.9; 3.41 vs 2.07; *P *< .001).

**Table 1:** Characteristics of study participants (n = 428) and their parents.

Characteristic		Participant	Father	Mother
Age (years), mean		15.7		
**Gender, n (%)**				
	Male	178 (41.6%)		
	Female	244 (57.0%)		
	No response	6 (1%)		
**Grade, n (%)**				
	9th	247 (57.7%)		
	10th	174 (57.7%)		
	No response	7 (2%)		
**Survey language, n (%)**				
	English	262 (61.2%)		
	Spanish	166 (38.8%)		
**Family structure, n (%)**				
	Both parents	176 (41.1%)		
	Mother	160 (37.4%)		
	Other	34 (37.3%)		
	No response	59 (14%)		
**US-born, n (%)**				
	Yes	150 (35.1%)		
	No	238 (55.6%)		
	No response	40 (9%)		
**How long in United States (years), n (%)**				
	0–3	112 (47.1%)		
	4–10	68 (29%)		
	10+	27 (11%)		
	No response	31 (13%)		
**Parent’s education level, n (%)**				
	Did not have any schooling		41 (10%)	48 (11%)
	Did not finish high school		73 (17%)	99 (23%)
	Graduated from high school		53 (12%)	59 (14%)
	Attended vocational/technical school		6 (1%)	15 (4%)
	Some college		17 (4%)	26 (6%)
	Graduated from college		35 (8%)	37 (9%)
	Not sure/don’t know/no response		203 (47.4%)	145 (33.9%)

**Table 2:** Utilization of mobile phone, Internet, and social media.

Media usage		n	%
**Mobile phone access**			
	Own a mobile phone	291	74.2%
	Have access to a mobile phone	64	16%
	Do not have access to a mobile phone	37	9%
**Reasons for mobile phone use**			
	Take pictures	316	90.6%
	Access the Internet	294	84.8%
	Send or receive instant messages	280	80.9%
	Play games	276	80.7%
	Send or receive email	23	68%
SMS^a ^use		334	94.4%
**Frequency of SMS use (messages/day)**			
	Low (1–20)	84	30%
	Medium (21–100)	79	29%
	High (>101)	113	40.9%
Internet access		385	98.0%
**Frequency of Internet use**			
	Daily	208	72.7%
	Less than daily	105	27.3%
**Reasons for Internet use**			
	School work	330	85.7%
	Email	299	81.7%
	Get health information	115	41.6%
Social networking account		384	97.2%
Mean number of social networking sites used		3.0 (range 0–8) (394)	
**Type of social networking account**			
	Facebook	342	89.1%
	YouTube	205	53.4%
	Twitter	196	51.0%
**Frequency of social networking use**			
	Daily	291	75.8%
	Less often	93	24%
**Reasons for use of social networking sites**			
	Post photos/videos	329	87.7%
	Post comments	329	86.4%
	Use instant messaging	318	86.0%
	Update status	320	85.6%
	Send private messages	273	73.0%
	Play games	166	46.6%

^a ^Short message service.

**Table 3:** Social media behaviors by gender, nativity, and survey language.

Behavior		Gender		Nativity		Survey language	
		Female (n = 244)	Male (n = 178)	US-born (n = 150)	Foreign-born (n = 238)	English (n = 262)	Spanish (n = 166)
Access to cell phone, n (%)		206 (90.8%)	143 (90.5%)	125 (91.2%)	195 (89.9%)	224 (93.3%)	126 (86.3%)*
**Frequency of SMS** ^a ^ **use (messages/day), n (%)**							
	Low (1–20)	45 (29%)	38 (33%)	27 (27%)	52 (35.9%)	42 (24%)	41 (43%)***
	Medium (1–100)	40 (26%)	36 (32%)	32 (31%)	38 (26%)	47 (27%)	30 (32%)***
	High (101+)	71 (46%)	40 (35%)	43 (42%)	55 (38%)	87 (49%)	24 (25%)***
Daily Internet use, n (%)		170 (76.2%)	105 (67.3%)*	107 (77.5%)	145 (68.7%)	183 (76.6%)	93 (66%)*
Social networking account, n (%)		224 (98.3%)	154 (95.7%)	137 (98.6%)	211 (96.4%)	239 (98.8%)	140 (94.6%)**
Facebook account, n (%)		196 (86.0%)	141 (87.6%)	125 (89.9%)	184 (84.0%)	220 (90.9%)	118 (79.7%)**
Mean number of social networking accounts		3.11	2.61**	3.41	2.54***	3.41	2.07***
Daily logging in to social networking account, n (%)		174 (77.7%)	113 (73.4%)	104 (75.9%)	154 (73.0%)	186 (77.8%)	102 (72.9%)

^a ^Short message service.

*P < .05, **P < .01, ***P < .001.

### Qualitative Phase

Staff and youth interviews yielded several themes regarding perceptions of an SMS and Facebook component to the ELYP program, including both positive and negative aspects of their use with Latino youth. The themes pulled from the qualitative data are divided into SMS- and Facebook-related themes separately.

#### Positive Aspects of an SMS Program Component

Staff and youth perceived SMS to have an overall positive impact because it would be an ideal way for staff and youth to become closer and to be more connected. One staff member stated that

some youth don’t really have adults, like positive role models, so especially a lot of them would express that they don’t do anything during the weekend, so I think it would be a nice comforting thing for them [to get a text message]. And then the reminder [message] would also obviously be a reminder and be helpful for them in that aspect, but I think overall, it’s that you are thinking about them.

When youth were asked what they had texted to staff in the past, their messages mainly concerned session times, checking whether a session had been cancelled, whether food was going to be available at a session, or other questions pertaining to group projects. While youth did not think it was necessary to have content-based reminder messages, staff thought motivational messages would be helpful reinforcements for youth.

#### Negative Aspects of an SMS Program Component

Staff expressed the two primary concerns for including an SMS component: (1) whether all youth participating in a program would have access to a mobile phone, and (2) that it could create the expectation that staff are available all hours of a day and every day. The first concern that not all youth have a mobile phone was noted by *both *staff and youth: “not all parents are on board with their students using cell phones, or using them all the time, or maybe even the idea of [staff] texting them.” Additionally, if “not every student has a cell phone...you might have a very effective tool, but if it doesn’t reach out to everyone it might alienate the other students.” The second concern, expressed by staff, would create an expectation that staff would be available at all hours of the day. If this were the case, one staff member discussed a possible scenario in which a student text messages a staff member when the student is in a crisis. Even if youth were told at the beginning of the program that the text messages were not to be used after hours or in times of emergency, one staff member stated that if “that kid texts and no one is there to respond, in some way I would feel bad.” One staff member commented that youth should not be dependent on staff and so SMS should clearly be about logistics of the program or reinforcement of the program content and should not be personal in any way.

Overall, staff thought that youth would want to receive between 2 and 4 text messages a week from staff pertaining either to logistics of the program, or to the curriculum and motivational messages. Youth said that, while they thought the logistics reminder messages would be helpful, receiving curriculum messages would be “a little” annoying and “would be helpful, but it isn’t necessary.” Additionally, both staff and youth thought that after school and during the evenings were the best times to send or receive text messages.

When consulted about 1-way versus 2-way text messaging, the overwhelming majority of youth declared that they preferred 2-way text messaging. Some youth stated that they would want to be able to ask a question back if they received a text from the program, or to simply ask a question of the program in general. One youth stated

it would be kind of hard because you have to tell them, like “oh, I can’t make it” or “do I need to take something?” Then you don’t see them until like the next session and then you ask them this and that, so it’s easier if you can be like, “oh I got your text, its ok, whatever.”

#### Positive Aspects of a Facebook Component

There are four major concepts related to positive aspects of Facebook. First, according to *both *staff and youth, access is widespread and all youth have a Facebook page. One staff member commented that “even the ones who don’t have a cell phone definitely check it at their friends house or maybe their own house, because usually parents are definitely willing to buy a computer but not so much give them a cell phone.” Second, youth with a Facebook page reported checking their page daily, which was also noted by staff. The third concept is about ease of communication. Staff noted that “[Facebook] is the most consistent way that you could be able to communicate with [youth],” which in turn would keep them engaged in the program. Many staff mentioned that being able to post events and opportunities for youth to gain service learning hours, potential field trips, pictures from past activities, and videos of youth in the program would keep the youth engaged. Youth reported that they would Like or join a program-specific Facebook page, and they would also want to make comments on the page. The final positive aspect centered on the open access of Facebook, which provides youth the opportunity to be more honest and open about their thoughts and feelings, which according to staff not only has the potential of bringing a group of young people closer together, but also offers staff insight into the youth’s context.

#### Negative Aspects of a Facebook Component

The major concern among staff about a health program-specific Facebook page was the possibility that it would enable cyberbullying of youth who joined the page, and that youth would post inappropriate pictures, videos, or comments to the Facebook page if it was not highly regulated and controlled by the overseeing organization. In addition, staff noted that not every student would be happy with the program all the time, “so if they get really frustrated or if they get kicked out of the program because of attendance, they could express some of that” on the Facebook page; in short, it is “a platform for [youth] to lash out.” Additionally, while youth had responded that they would initially join a program-specific Facebook page, they stated that if they were unable to post comments or pictures, or have a personal interaction with the Facebook page, then they would not check it as often.

## Discussion

Harnessing new technology to reach youth with positive and healthy messages could be extremely powerful, and therefore it is critical to fully understand how adolescents from different communities and cultures use technology. This study found results comparable with those of the Pew Hispanic Center teen survey on mobile and social media use [12]. The Pew report found that the use of mobile technology greatly differs among Latino teens depending on nativity and language, with 65% of native-born teens saying they communicated with friends using text messaging versus 26% of foreign-born teens, and 68% of English-dominant and 50% of bilingual young Latinos using text messaging daily to communicate with friends, compared with only 19% of Spanish-dominant Latinos. Similar to that report, the present study found significant differences by survey language and nativity, with 93% of youth who took the survey in English versus 86% of youth who took it in Spanish having access to a cell phone. Further, 42% of US-born youth versus 38% of foreign-born youth had a higher frequency of SMS use, and 49% of those who took the survey in English versus 25% who took it in Spanish had a higher SMS usage frequency.

The Pew report also found that 23% of young Latinos used social media sites such as Facebook or MySpace daily to communicate with their friends, and native-born Latinos were more likely to use social media sites. Similarly, this study found that 97% had a social media account and 89% had a Facebook account. Further, 91% of youth who took the survey in English versus 80% who took it in Spanish had a Facebook account, and 90% of US-born youth versus 84% of foreign-born youth had a Facebook account.

Overall, the qualitative findings were very positive with respect to use of SMS and social media for public health interventions. Although several negative aspects were mentioned, most could be addressed through appropriate system design and use by program staff.

The study also found that Facebook is slightly more feasible than SMS as a method of communicating health messages due to issues surrounding lack of access to mobile phones and the youth participants’ overwhelming access to and usage of Facebook. Due to the majority of youth being non-US-born, even though they completed the survey in English, each component should be available in English and Spanish, and students should be able to opt in to either language.

The differences found in survey language indicate that Spanish-language respondents had significantly less access and less frequent use of social media. Furthermore, those who took the survey in Spanish were more likely to be recent immigrants, within the last 3 years. To address these significant and disadvantageous differences, programs must ensure that any social media component is available in both languages so as not to isolate the Spanish-speaking youth from this aspect of the intervention.

Since mobile phone access may be a financial issue, programs may need to expand efforts using free social media sites such as Facebook, although this does require access to a computer. Our findings indicate that use of Facebook is extremely common and may be particularly useful with youth who do not have access to SMS. If youth are simply unaware of these social media because of recent immigration, they can be encouraged to sign up for an account, or alternative strategies will need to be developed to reach these adolescents with the same information.

Although students expressed the desire to have 2-way messages, it may be more feasible for public health programs to have 1-way messages in an effort to not overwhelm staff. Youth need to be educated about the use of SMS and social media within the context of a program to increase their understanding that the program is sending them information and is not a 24-hour service, nor a means to contact the organization if there is a crisis. Rather, youth should be given phone numbers for local organizations that are equipped to handle crises such as 911, a local teen suicide hotline, and other local organizations as necessary to avoid liability issues.

### Strengths and Limitations

This study focused on a new and growing youth population in the United States with significant public health needs. However, there are a few limitations of the current study. This was a cross-sectional sample of youth and therefore it is not possible to examine causal relationships between nativity or language use and SMS or social media behavior. Additionally, although nativity and language are frequently used as proxy measures for acculturation, future studies should use more salient and robust measures that capture the complex and dynamic process of acculturation. Finally, this study comprised a subsample of youth participating in a research and demonstration project (ELYP) and may not be fully representative of a national sample of Latino youth. However, this sample consisted of Latino youth from Central America, primarily El Salvador and Peru, which is representative of the growing Latino population in Maryland. Further, future studies ought to examine cultural and geographic differences around SMS and social media use, as there are certainly distinct characteristics and traditions between Latino subgroups.

### Conclusions

SMS and social media are pervasive among Latino youth, and program staff and youth perceive these as credible and essential methods of communication in the context of public health programs. In fact, the frequency of use is so significant that public health interventions must continue to innovate and maximize new ways to reach young people to reinforce public health messages and education. Furthermore, given the differences found in this study by nativity and survey language, future research must delve deeper to understand differences in utilization and behavior by acculturation and other cultural factors such as parenting and family influences. Given the substantial role of parenting and family influences on adolescents living in immigrant families, and these differences by acculturation, future research must explore how these factors may mediate SMS and social media utilization and behavior.

## Multimedia Appendix 1

Quantitative survey administered to Latino youth.

## Multimedia Appendix 2

Key informant field guide for staff working with Latino youth.

## Multimedia Appendix 3

Triad field guide for Latino youth.

## Abbreviations

ELYP: Empowering Latino Youth Program
SMS: short message service
