# Supplementary material for: Public Health Interventions: Reaching Latino Adolescents via Short Message Service and Social Media
Source: J Med Internet Res. 2012 Jul 12;14(4):e99. doi: 10.2196/jmir.2178 (PMC3409615; doi:10.2196/jmir.2178)
Supplement: Supplementary file 1 [file jmir_v14i4e99_app1.pdf]

Empowering Latino Youth Survey

Thank you for participating in our survey. Your participation is very important. The information you give will be used to develop better youth development programs for teens.

Please answer the questions as honestly as possible based on what you really think or do. There are no right or wrong answers.

If you run into problems please ask the assistant for help. Each question will be read to you if you click on the play button after the question. To increase the volume press “F9”. To decrease the volume press “F8”.

Some questions are longer and require you to scroll to the bottom. You can use the "up" and "down" arrows or the mouse pad to scroll up and down on the right side of the screen - just like reading a website.

After you've clicked on a choice, click NEXT to be taken to the next screen.

If you decide you want to change one of your answers, just use the BACK button to go back.

If at any time you want to clear all your answers on a page click the RESET button.

Select NEXT to go to the next screen.

The computer has headphones to keep your answers private. Your teachers and your parents will not know your answers.

Completing the survey is voluntary, meaning you do not have to participate if you do not want to. You are also free to refuse to answer any of the questions. Whether or not you answer the questions will not affect anything in school or in this program.

Please feel free to ask our staff any questions you may have about this survey.

THANK YOU VERY MUCH FOR YOUR HELP!

Please let the assistant know if you have any questions.

In what language did you choose to take this survey? {G:\Audio Files\english audio\English baseline spring 2012 audio\2.wma}

- q Spanish
- q English

We would like to start by asking you some general questions about you and your family. Remember, all of your responses are private and confidential.

What is your gender?

- q Male
- q Female

What grade are you in?

- q 9th
- q 10th

What month were you born?

- q January
- q February
- q March
- q April
- q May
- q June
- q July
- q August
- q September

q October
q November
q December

What day were you born?

q 1
q 2
q 3
q 4
q 5
q 6
q 7
q 8
q 9
q 10
q 11
q 12
q 13
q 14
q 15
q 16
q 17
q 18
q 19
q 20
q 21
q 22
q 23
q 24
q 25
q 26
q 27
q 28
q 29
q 30
q 31

What year were you born?

q 1990
q 1991
q 1992
q 1993
q 1994
q 1995
q 1996
q 1997
q 1998
q 1999
q 2000

In what country were you born?

____

If not born in the United States, how long have you lived in the United States?

____

How many people live in your household <u>including you</u>?

____

Who do you live with in your household? Please choose all that apply

q Mother	q Foster Mother	q Parent's partner, boyfriend or girlfriend	q Other relative
q Father	q Foster Father	q Brother or Sister	q I live alone
q Stepfather	q Adoptive Mother	q Grandparent	q Other
q Stepmother	q Adoptive Father	q Aunt or Uncle	

Other

Please mark the highest level your mother completed in school

q she did not have any schooling	q she attended vocational/technical school	q don't know
q she did not finish high school	q she had some college	
q she graduated high school/GED	q she graduated college	

Please mark the highest level your father completed in school

q he did not have any schooling	q he attended vocational/technical school	q don't know
q he did not finish high school	q he had some college	
q he graduated high school/GED	q he graduated college	

The next few questions ask about your use of a cell phone, internet, and social networking sites.

Do you have a cell phone?

q Yes, I have my own cell phone
q No, I do not have my own cell phone **AND** I don't have access to one
q No, I do not have my own cell phone, **BUT** I borrow and/or share one

Do you use a cell phone to do any of the following things. Do you ever use a cell phone to...

	Yes	No
Send or receive email	q	q
Take a picture	q	q
Play music	q	q
Send or receive instant messages	q	q
Record a video	q	q
Play a game	q	q
Access the internet	q	q

Do you use text messaging?

- q Yes
- q No

Do you have unlimited text messaging?

- q Yes
- q No
- q Not sure

On an average day, would you say you send or receive...

- q 1-10 text messages per day
- q 11-20 per day
- q 21-50 per day
- q 51-100 per day
- q 101-200 per day
- q More than 200 text messages per day

How often do you send or receive text messages on your cell phone with the following people...

	Several times a day	At least once a day	A few times a week	Less often or never
Your friends	q	q	q	q
Your parents or guardians	q	q	q	q
Your brothers, sisters, or other family members	q	q	q	q
Your boyfriend or girlfriend	q	q	q	q

Do you use a computer at school, at home, or anywhere else at least sometimes?

- q Yes
- q No

Do you use the internet sometimes?

- q Yes
- q No

About how often do you use the internet or email?

- q Several times a day
- q About once a day
- q 3-5 times a week
- q 1-2 times a week
- q Less than once a week
- q Never

Do you ever use the internet to do any of the following things? Do you ever use the internet to...

	Yes	No
Send or read email	q	q
Look for health information online	q	q
Get information for homework or school projects	q	q

On which social networking site or sites do you have an account? (Remember, a social networking site is an online site that focuses on building and reflecting social relations among people who share interests and/or activities). Check all that apply

- q Facebook
- q MySpace
- q Twitter
- q Yahoo
- q YouTube
- q My yearbook
- q Tumblr
- q Google buzz
- q Flickr
- q UStream
- q Other
- q Don't have my own profile or account on a social networking site

Other

Now we'd like to know some of the specific ways you use social networking sites. Do you ever...

	Yes	No

Post comments to something a friend has posted	q	q
Send private messages to a friend within the site	q	q
Send instant messages or chat with a friend through the site	q	q
Tag people in posts, photos or videos	q	q
Post a status update	q	q
Post photos or videos	q	q
Play games on the site	q	q

About how often do you visit social networking sites?

q Several times a day
q About once a day
q 3 to 5 days a week
q 1 to 2 days a week
q Every few weeks
q Less often
