# Supplementary material for: Public Health Interventions: Reaching Latino Adolescents via Short Message Service and Social Media
Source: J Med Internet Res. 2012 Jul 12;14(4):e99. doi: 10.2196/jmir.2178 (PMC3409615; doi:10.2196/jmir.2178)
Supplement: Supplementary file 2 [file jmir_v14i4e99_app2.pdf]

Key Informant Field Guide: Staff

Introduction: Welcome, Overview, & Topic

Thank you for taking the time to meet with me today. My name is _________, and I will be facilitating our discussion today. With me is ________ who will be taking notes. We are both students at The George Washington University's School of Public Health and Health Services. Today, I am speaking to you to get your ideas on social media, how it can be used within an after school program, and what your thoughts are on specific methods of communication with youth such as Facebook and text messaging. Right now we are in the beginning stages of planning, so this discussion will not affect what your job responsibilities are.

1. Were you hired specifically as a Youth Development Worker?

<u>Probe:</u> Have you worked with adolescents before?

I would like to ask you a few questions about text messaging. Just so we are clear, do you know what text messaging is? (If not: Text messaging is when written messages are sent and received using mobile phones).

2. So with that, do you text message?

<u>Probe:</u> Who do you normally text message? In general, how often do you text? Everyday?
<u>Probe:</u> Do you ever text messages your colleagues at Identity? What do you normally text them about?
<u>Probe:</u> Do you ever text youth that you work with in the Identity programs? Tell me about that.
<u>Probe:</u> What do youth text you about?

3. Do you think text messaging could be used as part of the Be Yourself (TPP) program? Why or Why not?

<u>Probe:</u> How do you think it can be used? (reminders, curriculum messages etc.)
<u>Probe:</u> How many messages per week do you think should be sent to each youth? How much is too much?
<u>Probe:</u> When do you think the youth would want to receive messages? (ie. Time of day/certain days a week)
<u>Probe:</u> Do you think it would be useful to use text messaging to remind youth of logistics issues, such as session times and when consent forms are due?

4. What do you think the impact of this communication will be on the youth?

Probe: Can you think of the positive and/or negative impacts?

So we are going to switch gears a little bit now and talk about Facebook and how it could be used in the TPP program. Do you know what Facebook is?

5. How do you think social networking sites like Facebook could be used for the TPP program?

Probe: Do you think the youth would use a TPP Facebook page?

Probe: What would be the positive and negative reasons to using facebook?

Probe: Do you see any potential liability issues being connected to youth through Facebook?

Probe: Would you want to be responsible for posting to a Facebook page?

Probe: What kind of information do you think should be posted/passed to youth through Facebook?

6. There has been thought that Facebook could possibly be used to send messages to youth's accounts after they have finished the TPP program, what do you think about that?

Probe: Do you think youth would respond to the messages?

Probe: Can you think of other ways to communicate with youth after they have finished the program?

7. Is there anything else you would like to comment about Facebook or texting? Do you have any questions for me?

Thank you for your wonderful input. I will make sure to use your comments and suggestions when I am reworking the text messages to be used.
