# Supplementary material for: Public Health Interventions: Reaching Latino Adolescents via Short Message Service and Social Media
Source: J Med Internet Res. 2012 Jul 12;14(4):e99. doi: 10.2196/jmir.2178 (PMC3409615; doi:10.2196/jmir.2178)
Supplement: Supplementary file 3 [file jmir_v14i4e99_app3.pdf]

Triad Field Guide: Youth

Introduction: Welcome, Overview, & Topic

Thank you for taking the time to meet with me today. My name is __________, and I will be facilitating our discussion today. With me is ________ who will be taking notes. We are both students at The George Washington University's School of Public Health and Health Services. During this session the group is going to be asked to talk about social media, such as text messaging and Facebook, and how it can be used to enhance the program you all participated in this past Spring. There are no right or wrong answers and we simply want to know what you think works and what doesn't. Your participation in this focus group is voluntary, and you may stop your participation at any time. Participation will have no effect on any services you receive. We will be using the information from this group and other groups, however, no individual names will be obtained, and we will not tell anyone what was said by individuals in the group. What you say in here is confidential.

Our session will last for about 30 minutes but before we begin, let me remind you of some ground rules.

First some general guidelines:

- Respect everyone's opinions and let everyone be heard – only one person should talk at a time…we don't want to miss any of your comments.
- We're interested in the positive things you have to say and the negative things you may have to say about Identity and your experience here, so please be honest…we need to know everything. Sometimes the negative comments are what help a program get better.
- If you have any questions, do not hesitate to stop and ask us.
- You don't have to answer any questions you don't want to. You can stop your participation at any time. If you do choose to stop participating, we only ask that you not be disruptive. What you have to say is important to us.
- We're going to use first names this afternoon but when I write up the report, I won't use any of your names…so our talk is considered strictly confidential.

Let's begin.

1. **Does anyone here have a friend who doesn't have a cell phone?**
   Probe: What are some reasons why?
   Probe: What does that person do when they want to get in touch with someone?

2. **Does anyone here use text messaging?**

3. **Who do you usually text?**

4. **When during the day do you usually text?**

5. **How often do you usually text during the day?**
   Probe: What if you got a text from a number you didn't recognize, do you think you would look at it?

6. **Have you ever texted a staff member?**
   Probe: What kinds of things did you text them about? Was it something related to your being at a session? Did it have something to do with what you spoke about in session, or was it more personal questions?

7. **Would you want the staff members who run your program to text you during the week to remind you of sessions? Why?**
   Probe: Do you think this would be helpful in making sure you go to the sessions?
   Probe: Would you want the Identity staff text you during the week to remind you of what you learned during your sessions that week?

8. **What time of day would you like to receive text messages from staff? Are there times you think that are better than others?**

So there are different forms of text messaging called 1-way and 2-way text messaging. 1-way text messaging is exactly how it sounds, it only goes one way. For example I text you something, say a reminder that we have to meet, but you can't text me back. 2-way text messaging is how most people text using their cell phone. For example, you text someone to ask "how you are doing?" and then you receive a text back "I'm doing great!"

9. **What if the messages were only 1-way, as in the staff can only text you and you can't text back, would it bother you if you couldn't text back?**

We are going to switch gears now and talk a little about blogs and Facebook.

10. **Do you have a Facebook account?** How often do you check it?
    Probe: What do you do on Facebook? Post on you friend's walls?
    Probe: Do you send messages to friends using Facebook?
11. **Are you Facebook friends with other youth in the program? Or with any staff?**

12. Do you think Facebook is a good way to keep in touch other youth during the program?

Probe: What about after the program?
Probe: Would you join a Facebook page for this Identity program?
Probe: How often would you check the Facebook page?

13. Would you want to be able to post to the Facebook page? Why?

Probe: if you weren't able to post to the page, but Identity staff posted messages and other things, would you still look at it?

14. If someone from the program tried to contact you through Facebook in one year, would you respond? Why or why not?

15. Is there anything else you would like to comment about on Facebook or text messaging? Do you have any questions for me?

Thank you for your help with the messages.
